# Reduced Incidence of Pneumothorax and Chest Tube Placement following Transthoracic CT-Guided Lung Biopsy with Gelatin Sponge Torpedo Track Embolization: A Propensity Score–Matched Study

**DOI:** 10.3390/jcm13164666

**Published:** 2024-08-09

**Authors:** Sasikorn Feinggumloon, Panupong Radchauppanone, Tanapong Panpikoon, Chinnarat Buangam, Kaewpitcha Pichitpichatkul, Tharintorn Treesit

**Affiliations:** 1 Department of Diagnostic and Therapeutic Radiology, Faculty of Medicine, Ramathibodi Hospital, Mahidol University, Bangkok 10400, Thailand; 2 Chiangrai Prachanukroh Hospital, Chiang Rai 57000, Thailand

**Keywords:** percutaneous lung biopsy, computed tomography, gelatin sponge torpedo, track embolization, pneumothorax, chest tube placement

## Abstract

**Objectives**: To evaluate the effectiveness of track embolization using gelatin sponge torpedo in reducing the incidence of pneumothorax and chest tube placement after percutaneous CT-guided lung biopsy. **Methods**: A retrospective single-center analysis of percutaneous computed tomography (CT)-guided transthoracic lung biopsies was performed between 2017 and 2022. After excluding the patients who received an ultrasound-guided biopsy, combined lung biopsy with ablation, fiducial placement, unsuccessful procedure due to uncooperative patient, and age under 18 years, 884 patients’ clinical information was collected (667 without track embolization and 217 with track embolization). The incidence of early and late pneumothorax and chest tube placement were compared between the two groups. Propensity score matching (PSM) was applied to minimize selection bias. Univariable and multivariable analyses were performed to determine risk factors for pneumothorax. **Results**: After PSM, the baseline differences and all factors that could affect the incidence of pneumothorax were balanced between the track embolization group (217 patients) and the non-track embolization group (217 patients). The incidence rates of early pneumothorax (13.4% vs. 24.0% *p* = 0.005), late pneumothorax (11.0% vs. 18.0% *p* = 0.021), and chest tube placement (0.9% vs. 4.6% *p* = 0.036) were significantly decreased in the track embolization group. However, the success rate of tissue diagnosis yield and length of hospital stay were not significantly different between the two groups. In multivariate analysis, the risk of pneumothorax increased as the fissure was passed (OR = 3.719, *p* = 0.027). **Conclusions**: Using track embolization with a gelatin sponge torpedo significantly decreased the incidence of pneumothorax and chest tube placement following percutaneous CT-guided lung biopsy.

## 1. Introduction

The advancement of high-resolution computed tomography (CT) has significantly increased the detection of pulmonary nodules. Therefore, the imaging diagnosis remains challenging and requires pathological diagnosis for further management. CT-guided percutaneous transthoracic needle biopsy is a safe and effective procedure to obtain pulmonary lesions for diagnosis. The method is minimally invasive with high diagnostic accuracy, ranging between 74 and 95%, and a low mortality rate [1,2,3,4,5,6]. Nevertheless, the reported incidence of complications varies widely.

The pooled rate of pneumothorax for CT-guided core biopsy was 25.3%, with 5.6% of pneumothoraxes requiring intervention, 18.0% pulmonary hemorrhage, and 4.1% hemoptysis [7]. The majority of complications are pneumothorax, which can be managed conservatively with oxygen supplements. If the pneumothorax enlarges or becomes symptomatic, chest tube drainage and extended hospital stay are required. Therefore, reducing the incidence of pneumothorax following CT-guided lung biopsy could minimize morbidity and length of hospital stay.

Different techniques have been attempted to lower the incidence of pneumothorax by sealing the tract to prevent air from leaking into the pleural space [8]. 

Collagen foam plugs have been reported to reduce the incidence of pneumothorax by sealing the needle track [9]. However, the data acquired was limited to a small population. Although a hydrogel plug, which is a commercially available device, has been previously reported for closing the needle tract, it still comes with an increase in the overall cost of the procedure.

We hypothesized that using a gelatin sponge torpedo would have a lower pneumothorax and chest tube insertion rate. Therefore, the study aims to evaluate the effectiveness of track embolization using the gelatin sponge torpedo technique for reducing the incidence of pneumothorax and chest tube insertion. Additionally, the study aims to minimize selection bias by using propensity score matching (PSM).

## 2. Materials and Methods

Study population

This retrospective, single-center study was approved by the institutional review board. The consecutive series of 962 transthoracic biopsies in our institute from January 2017 to October 2022 was retrieved from the hospital database. After excluding the patients who received an ultrasound-guided biopsy, combined lung biopsy with ablation, fiducial placement, unsuccessful procedure due to uncooperative patient, and pediatric patients (age < 18 years) who were unable to follow the instructions, 884 patients’ clinical information was collected from the electronic medical records.

Demographic data

Electronic medical records were reviewed to obtain patient information, including age, sex, underlying lung disease, size and morphology of lesion, location, depth from the pleura, patient’s position, size of the coaxial needle, number of passing fissures, number of cutting attempts, total cutting length, pathological diagnosis, complication, and interventions. The CT images and chest radiographs were reviewed throughout the hospitalization to detect any complications or adverse events. 

Eight hundred eighty-four patients were allocated into two groups according to the application of track embolization using a gelatin sponge torpedo. The decision to perform track embolization was based on the operator’s preference. The number of patients in each group with or without track embolization was 667 (329 men (49%), 338 women (51%); mean age 63.95 years) and 217 (97 men (45%), 120 women (55%); mean age 64.94 years), respectively. Between these two groups, they showed significant differences in depth from pleura (*p* = 0.01), morphology of the lesion (*p* = 0.019), size of coaxial needle (*p* = 0), and total cutting length (*p* = 0.015), while other variables showed no significant difference. The patient and procedural characteristics are summarized in Table 1. The presence of pneumothorax following the procedure detected on chest radiographs within 4 h and between 4 and 24 h were compared between the two groups. The presence of post-biopsy chest tube placement was reviewed from the medical records. Additional complications such as perilesional hemorrhage, hemothorax, and hemoptysis requiring intubation were collected, along with the diagnostic yield and length of hospital stay after the procedure.

Procedure technique

**Figure 1:**
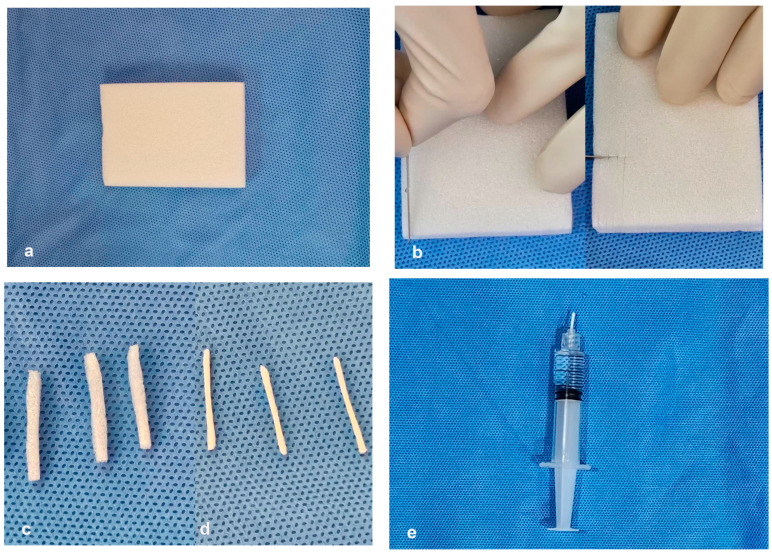
The preparation of the gelatin sponge torpedo. (**a**) A sheet of gelatin sponge was cut (**b**,**c**) with a diameter of 1–2 mm, 20 mm in length, and (**d**) molded into a torpedo form. (**e**) The gelatin sponge torpedo was loaded into a 5 mL Luer lock syringe containing normal saline solution.

**Figure 2:**
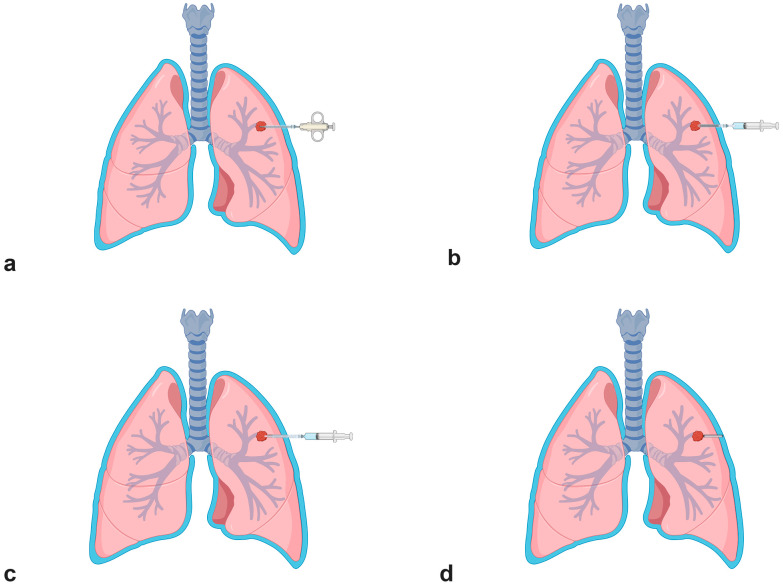
(**a**) Gelfoam torpedo was inserted into the coaxial needle. (**b**) The syringe plunger was depressed to deploy the torpedo while simultaneously (**c**) withdrawing the coaxial needle. (**d**) The number of torpedoes was determined by the length of the needle path from the lesion to the pleura.

All transthoracic biopsies were performed under CT guidance by an interventional radiologist, with 5–12 years of experience or a fellowship-trained radiologist under the supervision of the attending physician. The pre-procedural images were reviewed, and the procedures were performed using Canon Alphenix Hybrid Angio-CT, Canon Medical Systems Corporation, Tochigi, Japan or Phillips Brilliance iCT 256, Philips Medical Systems (Cleveland), Inc., Cleveland, OH, USA. The risks, benefits, and potential procedural-related complications were explained, and informed consent was obtained before the procedure. The patient was positioned in a supine, decubitus, or prone posture, and the skin was cleaned using an aseptic technique. Local anesthesia was injected with or without conscious sedation. Transthoracic biopsy was performed with coaxial technique using an 18-gauge semi-automatic biopsy needle (BARD^®^ MISSION^®^ Disposable Core Biopsy Instrument with 17 G coaxial needle, Medax Bio-Feather Semi-Automatic Spring-Loaded Biopsy with 16 G coaxial needle, TemnoTM biopsy needle Merit Medical system with 17 G coaxial needle, and Semi-automatic DSX Tsunami medical S.r.l with 16 G coaxial needle) under CT guidance. After adequate tissue was obtained, the coaxial needle was withdrawn in the non-track embolization group. In the track embolization group, a gelatin sponge sheet (Spongostan, Ethicon™, Inc., Somerville, NJ, USA) was prepared by cutting into rectangular shapes with a diameter of 1–2 mm, 20 mm in length, and molded into a torpedo form. Then, a gelatin sponge torpedo was loaded into a 5 mL Luer lock syringe containing normal saline solution (Figure 1). Then, the syringe plunger was depressed to deploy the torpedo while simultaneously withdrawing the coaxial needle. The number of torpedoes delivered was determined by the length of the needle path from the lesion to the pleura (Figure 2). 

**Figure 3:**
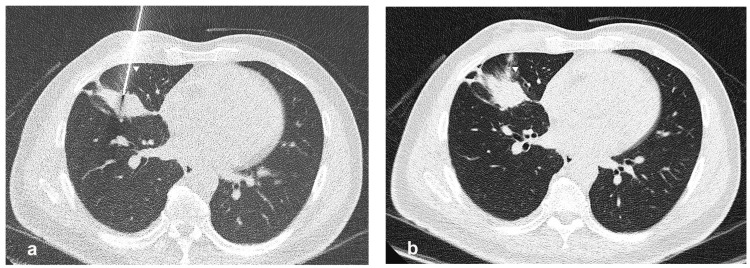
(**a**) Axial CT scan shows the pulmonary lesion at the right middle lobe with the 17-gauge coaxial biopsy needle (arrowhead) in the lesion. (**b**) Post-biopsy CT scan performed after track embolization with gelatin sponge torpedo showed a radiolucent track along the needle path (arrowhead).

All patients underwent post-procedural immediate CT (Figure 3); if there was no progressive or asymptomatic pneumothorax, the patient was transferred to the observation ward, followed by a chest radiograph within 4 h to detect early pneumothorax and on the next day after the procedure for late pneumothorax. When a progressive or symptomatic pneumothorax developed, the patient was treated with simple aspiration or chest tube insertion. The patient was discharged if the symptoms or pneumothorax were resolved.

Statistical analysis

The statistical analysis was performed using STATA, version 17 (StataCorp, College Station, TX, USA). A Chi-square or Fisher exact test was used to compare the differences between the categorical variables. The differences in quantitative variables were compared using an independent sample T-test. As the two groups were not randomly assigned, the effect of confounding factors was reduced by generating propensity score matching prior to calculating the treatment effect. The propensity model included various independent variables that might have affected post-biopsy pneumothorax and chest tube placement. The logit function was used to calculate the likelihood of being assigned to each group based on these variables, including age, underlying lung disease, lesion morphology, depth from the pleura, size of the coaxial needle, passing fissure count, cutting attempts, and total cutting length. The calculated propensity was subsequently used in the final model as a covariate to adjust for confounding by indication and contraindication. We used a one-to-one matching method to balance the chosen variables between the track embolization and non-track embolization groups based on the computed scores and then showed histograms of the propensity score distributions of both groups to demonstrate the balance of the score before and after matching.

**Figure 4:**
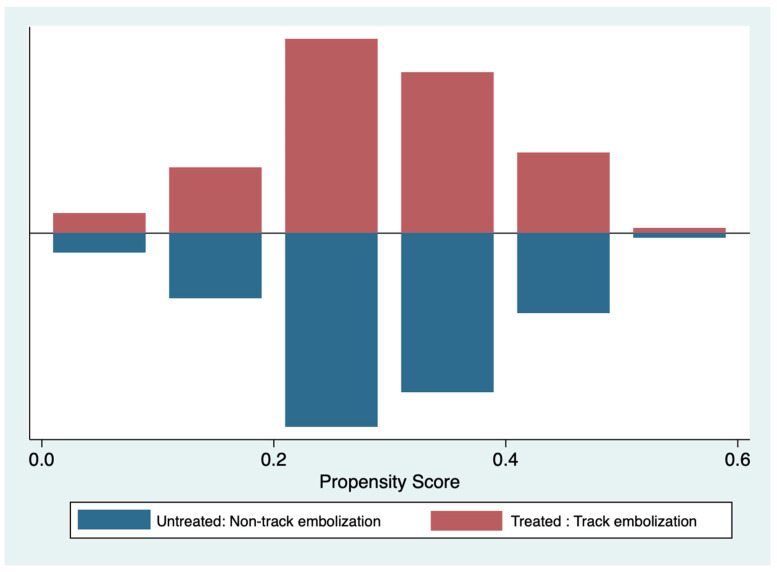
Matching graph of the propensity score before and after matching.

After adjusting the propensity score, the baseline characteristics were reanalyzed and compared between the two groups using the Chi-square or Fisher exact test and independent sample T-test. The standardized differences before and after matching were estimated, and the distribution of propensity scores was plotted (Figure 4). The incidence of early and delayed pneumothorax, chest tube placement, diagnostic yield, and length of hospital stay were compared between the two groups using the Chi-square or Fisher exact test. The predictive factors, including age, sex, underlying lung disease, lesion morphology, lesion location, maximal diameter of the lesion, depth from the pleura, patient position during the procedure, size of coaxial needle, passing fissure count, cutting attempts, and total cutting length for pneumothorax were analyzed using univariate and multivariate logistic regression analysis. Odds ratios (OR) and 95% confidence intervals were calculated. Two-sided *p* values less than 0.05 were considered statistically significant differences.

## 3. Results

After propensity score matching, the sample size was reduced from 884 to 434 by removing 450 patients from the non-track embolization group. Finally, there were 217 patients in each group. There was no significant difference in the baseline characteristics of the patients and lesions after propensity score matching, as shown in Table 2.

### 3.1. Comparison of Pneumothorax

Within 4 h following the procedure, early pneumothorax was observed in 52 (24.0%) patients in the non-track embolization group. On the other hand, among the patients in the track embolization group, 29 (13.4%) patients developed pneumothorax, which was significantly lower compared to the non-track embolization group (*p* = 0.005). 

At 4–24 h after the procedure, the incidence of late pneumothorax was also significantly lower in track embolization group with 23 (10.6%) patients compared to the non-track embolization group with 40 (18.4%) patients (*p* = 0.021) (Table 3).

### 3.2. Chest Tube Insertion Rate

The rate of chest tube insertion was significantly lower in the track embolization group (2/217, 0.9%) than in the non-track embolization group (10/217, 4.6%) (*p* = 0.036) (Table 3).

There were no significant differences in diagnostic yield (95.9% in the non-track embolization group vs. 97% in the track embolization group) and length of hospital stay between these two groups (2.6 ± 5.8 days in the non-track embolization group vs. 3.4 ± 10.7 days in the track embolization group).

### 3.3. Variable Factors Affecting Pneumothorax

The results of univariate analysis showed that the risk factors for pneumothorax within 4 h after the procedure were the small size of the lesion (*p* = 0.0045) and the small diameter of the lesion measured in the needle trajectory path (*p* = 0.029) (Table 4).

In multivariate analysis, only one predicting factor that affected the risk of pneumothorax was passing the fissure (OR 3.72, 95% CI 1.16–11.89, *p* = 0.027). There were no significant differences observed in the other potential risk factors, such as underlying emphysematous lung with OR 0.62 (95% CI 0.18–2.14, *p* = 0.533), lesion diameter with OR 0.97 (95% CI 0.93–1.00, *p* = 0.057), depth from pleura with OR 1.0 (95% CI 0.98–1.02, *p* = 0.9), subsolid morphology with OR 3.23 (95% CI 0.90–11.68, *p* = 0.073), ground glass morphology with OR 1.68 (95% CI 0.38–7.51, *p* = 0.496, 17 G coaxial needle with OR 1.26 (95% CI 0.30–5.29, *p* = 0.755), and total cutting length with OR 1.13 (95% CI 0.95–1.34, *p* = 0.18).

## 4. Discussion

This study reveals the advantages of track embolization using a gelatin sponge torpedo, regardless of whether propensity score matching analysis is used to minimize potential confounding factors and selection bias. The incidence of pneumothorax and chest tube placement after percutaneous biopsy under CT guidance was significantly lower in the track embolization group than in the non-track embolization group.

Pneumothorax is the most common complication that occurs following a percutaneous lung biopsy. The pooled rate of pneumothorax was 25.3% and 5.6% of pneumothorax incidents required intervention [7,10]. These pneumothorax and chest tube insertion rates are lower than the suggested quality improvement (QI) thresholds recommended by the Society of Interventional Radiology (SIR), which are 45% and 20%, respectively [11]. Nevertheless, the rates remain higher than the recommended guidelines established by the British Thoracic Society (BTS) and the cardiovascular and interventional CIRSE, which reference 20.5% and 18.8–25.3%, respectively. The incidence of pneumothorax requiring chest tube drainage was reported based on BTS standards and 4.3–5.1% according to the cardiovascular and interventional CIRSE guidelines [1,10,11].

A systemic review and meta-analysis demonstrated that several techniques could effectively reduce the incidence of pneumothorax and chest tube insertion rates [8]. These techniques include using normal saline track sealant, blood patch, hydrogel plug, and gelatin sponge slurry [12,13,14,15,16,17,18,19,20,21,22,23,24]. Among these, using normal saline track sealant proved to be the most effective technique for reducing the occurrence of pneumothorax and pneumothoraxes requiring chest tube drainage. The recent study by Bourgeais et al. reported the incidence of pneumothorax in the normal saline instillation group at 19.4% compared with 40.9% in the non-instillation group [14]. The benefit of using normal saline sealing is that it is low-cost, has no adverse reaction, and is readily available. However, it is crucial to consider that the effectiveness of normal saline track sealant may vary depending on the amount of saline used, procedural technique, and operator’s expertise.

Alternative sealants and other techniques proved to be effective in reducing the occurrence of pneumothorax. One of the cost-effective, absorbable, and ready-to-use embolic materials is a gelatin sponge. The advantage of a water-insoluble hemostatic agent composed of porcine collagen is that it can be used in non-vascular and vascular procedures to provide mechanical tamponade and activate the intrinsic coagulation cascade. The gelation sponge was first reported to be used in slurry preparation in a study conducted by Tran et al. The study demonstrated a decrease in the pneumothorax rate from 10.7% to 6.9% [21]. However, it failed to show a significant difference in chest tube insertion between the track embolization group (6.9%) and the non-track embolization group (10.7%). Baadh et al. also demonstrated a significant reduction in the incidence of pneumothorax in the track embolization group compared to the non-track embolization group, with rates of 8.8% and 21%, respectively [22]. However, there was no difference in the need for post-procedural chest tube placement between the two groups. Two later publications demonstrated the significantly decreased pneumothorax and chest tube insertion rates [23,24]. Renier et al. showed a decrease in both pneumothorax rate (25.8% vs. 10%) and chest tube placement (12.2% vs. 3.5%) when using a 19 G coaxial needle followed by track embolization [23]. Grange et al. used 18 G needle biopsy and found a significantly lower incidence of both pneumothorax (44.5% vs. 27.1%) and chest tube placement (6.0% vs. 2.6%) as compared to the non-track embolization group [24]. Even though gelatin sponge has been previously reported to be used in slurry preparation in previous research studies, there is only one study by Engeler et al. using a compressed collagen foam plug preparation to occlude the needle track in percutaneous lung biopsy [9]. The incidence of pneumothorax in the track embolization group was lower compared to the non-track embolization group (8% vs. 28%). However, there was no significant change in the rate of chest tube placement. Additionally, there are two reports of track embolization using gelatin sponge torpedo following radiofrequency ablation [25,26]. Both pneumothorax and chest tube placement rates were significantly lower in the track embolization group despite the fact that the coaxial radiofrequency ablation needle (14–15 gauge) was larger than the biopsy needle (18–20 gauge).

In our cohort, after propensity score matching, the incidence of post-biopsy pneumothorax within 4 h was 18.7% (24.0% in the non-track embolization group and 13.4% in the track embolization group), and the incidence of delayed pneumothorax was 14.5% (18% in the non-track embolization group and 11% in the track embolization group). The incidence of chest tube insertion was 2.8% (4.6% in the non-track embolization group and 0.9% in the track embolization group). Our results on the incidence of both early and delayed detection of pneumothorax are consistent with studies that have used normal saline instillation or gelatin sponge slurry embolization. Meanwhile, our study observed a lower chest tube placement rate than other methods without a significant difference in diagnostic yield and length of hospital stay. Regarding the possibility of migration risk of embolic materials into the pulmonary veins and subsequent entry into the systemic circulation [27], no embolic complications were observed in our study. Moreover, the utilization of the gelatin sponge torpedo technique could potentially decrease this risk as compared to the gelatin sponge slurry because of its capacity to expand upon rehydration and conform to the needle track pathway, as visualized in immediate post-procedural CT scans in most of our cases (Figure 3).

In this study, the diagnostic yield did not differ between the two groups due to the similarity in coaxial needle size, cutting length, and number of cutting attempts. Despite a significant difference in the incidence of pneumothorax and chest tube placement, the duration of hospital stay was no different as every patient was admitted for observation and following chest radiograph, regardless of the presence of pneumothorax.

There were some limitations in this research. First, a single-center retrospective study may have residual or unmeasured confounders affecting the outcomes. Second, the difference in procedural technique, skill, and expertise among interventional radiologists can potentially impact the outcomes and results. Third, the decision to place the chest tube depends on the primary operator. Lastly, our analysis did not exclude cases that developed pneumothorax before the withdrawal of the biopsy needle, which could have affected the pneumothorax rate. Future studies need a large, multi-center study with standardized protocols to minimize bias. In addition, excluding cases with intra-procedural pneumothorax could provide a more accurate result. 

## 5. Conclusions

In conclusion, performing track embolization using a gelatin sponge torpedo after percutaneous lung biopsy can be an alternative material that is cost-effective, easy to use, and readily available to lower pneumothorax incidence and chest tube placement.

## Figures and Tables

**Table 1:** Demographic and baseline characteristics of the patients between the two groups.

	Non-Track Embolization Group (*n* = 667)	Track Embolization Group (*n* = 217)	*p*-Value	STD
Age (years)	64.0 ± 12.7	64.9 ± 12.8	0.318	0.08
Sex (n,%)			0.236	0.09
Male	329 (49)	97 (45)		
Female	338 (51)	120 (55)		
Underlying lung disease (n,%)			0.844	0.08
None	528 (79)	178 (82)		
Effusion	53 (8)	16 (7)		
Emphysematous lung	42 (6)	12 (6)		
Reticular fibrosis	44 (7)	11 (5)		
Morphology (n,%)				
Solid	472 (71)	172 (79)	0.019	0.16
Subsolid/Subgroundglass	44 (7)	6 (3)		
Ground glass opacity	12 (2)	8 (4)		
Cavity	28 (4)	6 (3)		
Consolidation	63 (9)	11 (5)		
Internal air bronchogram	48 (7)	14 (6)		
Location (n,%)			0.525	0.07
RUL	180 (27)	57 (26)		
RML	40 (6)	9 (4)		
RLL	160 (24)	46 (21)		
LUL	152 (23)	60 (28)		
LLL	132 (20)	45 (21)		
Maximal diameter (mm)	32.1 ± 21.0	32.9 ± 20.9	0.623	0.04
Maximal diameter from needle projection (mm)	27.3 ± 17.8	28.3 ± 16.6	0.469	0.06
Depth from pleura (mm)	13.8 ± 13.8	16.5 ± 13.2	0.01	0.2
Patient position, (n,%)			0.892	0.06
Supine	239 (36)	84 (39)		
Prone	326 (49)	103 (47)		
Right lateral decubitus	44 (7)	13 (6)		
Left lateral decubitus	58 (9)	17 (8)		
Coaxial needle (Gauge)			0	0.32
16	90 (13)	7 (3)		
17	574 (86)	210 (97)		
19	3 (0)	0 (0)		
Passing fissure			0.343	0.07
No	648 (97)	208 (96)		
Yes	19 (3)	9 (4)		
Number of cutting attempts	3.3 ± 1.3	3.2 ± 1.3	0.798	0.02
Total cutting length (cm)	5.7 ± 2.6	5.2 ± 2.3	0.015	0.2

Parametric data are presented as mean ± standard deviation and categorical data are presented as number (percentage). STD, standardized difference.

**Table 2:** Demographic and baseline characteristics of the patients between the two groups (propensity score–matched data).

	Non-Track Embolization Group (*n* = 217)	Track Embolization Group (*n* = 217)	*p*-Value	STD
Age (years)	64.4 ± 11.9	64.9 ± 12.8	0.7	0.00
Sex (n,%)			1.0	0.00
Male	97 (45)	97 (45)		
Female	120 (55)	120 (55)		
Underlying lung disease (n,%)			0.8	−0.1
None	185 (85)	178 (82)		
Effusion	13 (6)	16 (7)		
Emphysematous lung	11 (5)	12 (6)		
Reticular fibrosis	8 (4)	11 (5)		
Morphology (n,%)				
Solid	169 (78)	172 (79)	0.7	0.00
Subsolid/Subgroundglass	8 (4)	6 (3)		
Ground glass opacity	4 (2)	8 (4)		
Cavity	6 (3)	6 (3)		
Consolidation	17 (8)	11 (5)		
Internal air bronchogram	13 (6)	14 (6)		
Location (n,%)			0.6	0.00
RUL	54 (25)	57 (26)		
RML	17 (8)	9 (4)		
RLL	48 (22)	46 (21)		
LUL	54 (25)	60 (28)		
LLL	44 (20)	45 (21)		
Maximal diameter (mm)	28.9 ± 20.6	32.9 ± 20.9	0.1	−0.2
Maximal diameter from needle projection (mm)	25.3 ± 17.6	28.3 ± 16.6	0.1	−0.2
Depth from pleura (mm)	14.5 ± 14.0	16.5 ± 13.2	0.1	−0.1
Patient position, (n,%)			0.8	0.1
Supine	83 (38)	84 (39)		
Prone	97 (45)	103 (47)		
Right lateral decubitus	17 (8)	13 (6)		
Left lateral decubitus	20 (9)	17 (8)		
Coaxial needle (Gauge)			0.6	−0.1
16	10 (5)	7 (3)		
17	207 (95)	210 (97)		
Passing fissure			1.0	0.0
No	208 (96)	208 (96)		
Yes	9 (4)	9 (4)		
Number of cutting attempts	3.1 ± 1.3	3.2 ± 1.3	0.4	−0.1
Total cutting length (cm)	4.8 ± 2.3	5.2 ± 2.3	0.1	0.0

Parametric data are presented as mean ± standard deviation and categorical data are presented as number (percentage). STD, standardized difference.

**Table 3:** Incidence of pneumothorax and chest tube placement in both groups.

	Non-Track Embolization Group (*n* = 217)	Track Embolization Group (*n* = 217)	*p*-Value
Pneumothorax (n,%)			
Early (within 4 h)	52 (24)	29 (13)	0.001 *
Late (4 to 24 h)	40 (18)	23 (11)	0.009 *
Chest tube placement	10 (5)	2 (1)	0.036 *

Categorical data are presented as number (percentage); * Statistically significant difference at *p* < 0.05.

**Table 4:** Univariate analysis of risk factors for early pneumothorax.

	Univariate Analysis		
Variables	Odds Ratio	95%CI	*p* Value
Age (years)	1.00	0.98–1.02	0.69
Sex		1.32–3.59	0.89
Male	2.18		
Female	1.00		
Underlying lung disease			0.89
None	1.48	0.42–5.23	
Effusion	1.12	0.22–5.78	
Emphysematous lung	1.42	0.27–7.44	
Reticular fibrosis	1.00		
Morphology			0.27
Solid	2.83	0.65–12.37	
Subsolid/Subgroundglass	7.89	1.24–49.83	
Ground glass opacity	3.67	0.52–25.77	
Cavity	3.67	0.52–25.77	
Consolidation	1.00		
Internal air bronchogram	4.28	0.79–23.19	
Location			0.40
RUL	1.76	0.89–3.51	
RML	2.01	0.72–5.59	
RLL	1.26	0.60–2.65	
LUL	1.00		
LLL	1.07	0.49–2.33	
Maximal diameter (mm)	0.98	0.96–0.99	0.00 *
Maximal diameter from needle projection (mm)	0.98	0.96–1.00	0.03 *
Depth from pleura (mm)	1.01	1.00–1.03	0.12
Patient position			0.16
Supine	1.71	0.99–2.95	
Prone	1.00		
Right lateral decubitus	1.81	0.70–4.67	
Left lateral decubitus	2.02	0.85–4.82	
Coaxial needle (Gauge)			0.91
16	1.00		
17	1.08	0.30–3.91	
Passing fissure			0.06
No	1.00		
Yes	2.76	1.02–7.50	
Number of cutting attempts	0.97	0.80–1.17	0.73
Total cutting length (cm)	1.00	0.89–1.11	0.95

CI = confidence interval; * Statistically significant difference at *p* < 0.05.

## Data Availability

The data that support the findings of this study are available from the corresponding author upon reasonable request.

## References

[1] Manhire A., Charig M., Clelland C., Gleeson F., Miller R., Moss H., Pointon K., Richardson C., Sawicka E. (2003). Guidelines for radiologically guided lung biopsy. Thorax.

[2] Li Y., Du Y., Yang H., Yu J., Xu X. (2013). CT-guided percutaneous core needle biopsy for small (≤20 mm) pulmonary lesions. Clin. Radiol..

[3] Wang Y., Jiang F., Tan X., Tian P. (2016). CT-guided percutaneous transthoracic needle biopsy for paramediastinal and nonparamediastinal lung lesions: Diagnostic yield and complications in 1484 patients. Medicine.

[4] Drumm O., Joyce E.A., de Blacam C., Gleeson T., Kavanagh J., McCarthy E., McDermott R., Beddy P. (2019). CT-guided lung biopsy: Effect of biopsy-side down position on pneumothorax and chest tube placement. Radiology.

[5] Veltri A., Bargellini I., Giorgi L., Almeida P.A.M.S., Akhan O. (2017). CIRSE guidelines on percutaneous needle biopsy (PNB). Cardiovasc. Interv. Radiol..

[6] Zhu J., Qu Y., Wang X., Jiang C., Mo J., Xi J., Wen Z. (2020). Risk factors associated with pulmonary hemorrhage and hemoptysis following percutaneous CT-guided transthoracic lung core needle biopsy: A retrospective study of 1,090 cases. Quant. Imaging Med. Surg..

[7] Heerink W.J., de Bock G.H., de Jonge G.J., Groen H.J., Vliegenthart R., Oudkerk M. (2017). Complication rates of CT-guided transthoracic lung biopsy: Meta-analysis. Eur. Radiol..

[8] Huo Y.R., Chan M.V., Habib A.-R., Lui I., Ridley L. (2019). Post-biopsy manoeuvres to reduce pneumothorax incidence in CT-guided transthoracic lung biopsies: A systematic review and meta-analysis. Cardiovasc. Interv. Radiol..

[9] Engeler C.E., Hunter D.W., Castaneda-Zuniga W., Tashjian J.H., Yedlicka J.W., Amplatz K. (1992). Pneumothorax after lung biopsy: Pre-vention with transpleural placement of compressed collagen foam plugs. Radiology.

[10] Huo Y.R., Chan M.V., Habib A.-R., Lui I., Ridley L. (2020). Pneumothorax rates in CT-Guided lung biopsies: A comprehensive systematic review and meta-analysis of risk factors. Br. J. Radiol..

[11] Sheth R.A., Baerlocher M.O., Connolly B.L., Dariushnia S.R., Shyn P.B., Vatsky S., Tam A.L., Gupta S. (2020). Society of interventional radiology quality improvement standards on percutaneous needle biopsy in adult and pediatric patients. J. Vasc. Interv. Radiol..

[12] Billich C., Muche R., Brenner G., Schmidt S.A., Krüger S., Brambs H.-J., Pauls S. (2008). CT-guided lung biopsy: Incidence of pneumothorax after instillation of NaCl into the biopsy track. Eur. Radiol..

[13] Li Y., Du Y., Luo T., Yang H., Yu J., Xu X., Zheng H., Li B. (2015). Usefulness of normal saline for sealing the needle track after CT-guided lung biopsy. Clin. Radiol..

[14] Bourgeais G., Frampas E., Liberge R., Nicolas A., Defrance C., Blanc F.-X., Coudol S., Morla O. (2024). Pneumothorax Incidence with Normal Saline Instillation for Sealing the Needle Track After Computed Tomography-Guided Percutaneous Lung Biopsy. CardioVascular Interv. Radiol..

[15] Wagner J.M., Hinshaw J.L., Lubner M.G., Robbins J.B., Kim D.H., Pickhardt P.J., Lee F.T. (2011). CT-guided lung biopsies: Pleural blood patching reduces the rate of chest tube placement for postbiopsy pneumothorax. Am. J. Roentgenol..

[16] Malone L.J., Stanfill R.M., Wang H., Fahey K.M., Bertino R.E. (2013). Effect of intraparenchymal blood patch on rates of pneumothorax and pneumothorax requiring chest tube placement after percutaneous lung biopsy. Am. J. Roentgenol..

[17] Clayton J.D., Elicker B.M., Ordovas K.G., Kohi M.P., Nguyen J., Naeger D.M. (2016). Nonclotted blood patch technique reduces pneumothorax and chest tube placement rates after percutaneous lung biopsies. J. Thorac. Imaging.

[18] Türk Y., Devecioğlu İ. (2021). A retrospective analysis of the effectiveness of extrapleural autologous blood patch injection on pneumothorax and intervention need in CT-guided lung biopsy. CardioVascular Interv. Radiol..

[19] Zaetta J.M., Licht M.O., Fisher J.S., Avelar R.L., Group B.-S.S. (2010). A lung biopsy tract plug for reduction of postbiopsy pneumothorax and other complications: Results of a prospective, multicenter, randomized, controlled clinical study. J. Vasc. Interv. Radiol..

[20] Ahrar J.U., Gupta S., Ensor J.E., Mahvash A., Sabir S.H., Steele J.R., McRae S.E., Avritscher R., Huang S.Y., Odisio B.C. (2017). Efficacy of a self-expanding tract sealant device in the reduction of pneumothorax and chest tube placement rates after percutaneous lung biopsy: A matched controlled study using propensity score analysis. Cardiovasc. Interv. Radiol..

[21] Tran A.A., Brown S.B., Rosenberg J., Hovsepian D.M. (2014). Tract embolization with gelatin sponge slurry for prevention of pneumothorax after percutaneous computed tomography-guided lung biopsy. Cardiovasc. Interv. Radiol..

[22] Baadh A.S., Hoffmann J.C., Fadl A., Danda D., Bhat V.R., Georgiou N., Hon M. (2016). Utilization of the track embolization technique to improve the safety of percutaneous lung biopsy and/or fiducial marker placement. Clin. Imaging.

[23] Renier H., Gérard L., Lamborelle P., Cousin F. (2020). Efficacy of the tract embolization technique with gelatin sponge slurry to reduce pneumothorax and chest tube placement after percutaneous CT-guided lung biopsy. Cardiovasc. Interv. Radiol..

[24] Grange R., Di Bisceglie M., Habert P., Resseguier N., Sarkissian R., Ferre M., Dassa M., Grange S., Izaaryene J., Piana G. (2023). Evaluation of preventive tract embolization with standardized gelatin sponge slurry on chest tube placement rate after CT-guided lung biopsy: A propensity score analysis. Insights Into Imaging.

[25] Izaaryene J., Mancini J., Louis G., Chaumoitre K., Bartoli J.-M., Vidal V., Gaubert J.-Y. (2017). Embolisation of pulmonary radio frequency pathway–a randomised trial. Int. J. Hyperth..

[26] Graveleau P., Frampas É., Perret C., Volpi S., Blanc F.-X., Goronflot T., Liberge R. (2024). Chest tube placement incidence when using gelatin sponge torpedoes after pulmonary radiofrequency ablation. Res. Diagn. Interv. Imaging.

[27] de Baère T. (2021). Pneumothorax and lung thermal ablation: Is it a complication? Is it only about tract sealing?. Cardiovasc. Interv. Radiol..

